# Evaluation of an Automated Choroid Segmentation Algorithm in a Longitudinal Kidney Donor and Recipient Cohort

**DOI:** 10.1167/tvst.12.11.19

**Published:** 2023-11-17

**Authors:** Jamie Burke, Dan Pugh, Tariq Farrah, Charlene Hamid, Emily Godden, Thomas J. MacGillivray, Neeraj Dhaun, J. Kenneth Baillie, Stuart King, Ian J. C. MacCormick

**Affiliations:** 1 School of Mathematics, University of Edinburgh, College of Science and Engineering, Edinburgh, UK; 2 University/BHF Centre for Cardiovascular Science, The Queen's Medical Research Institute, University of Edinburgh, Edinburgh, UK; 3 Imaging Facility, University of Edinburgh, The Queen's Medical Research Institute, Edinburgh, UK; 4 Emergency Department, Royal Infirmary of Edinburgh, Edinburgh, UK; 5 Centre for Clinical Brain Sciences, University of Edinburgh, Edinburgh, UK; 6 Deanery of Clinical Sciences, University of Edinburgh, College of Medicine and Veterinary Medicine, Edinburgh, UK; 7 Centre for Inflammation Research, The Queen's Medical Research Institute, University of Edinburgh, Edinburgh, UK; 8 Institute for Adaptive and Neural Computation, School of Informatics, University of Edinburgh, Edinburgh, UK

**Keywords:** choroid, optical coherence tomography, renal transplantation, choroidal thickness, image segmentation

## Abstract

**Purpose:**

To evaluate the performance of an automated choroid segmentation algorithm in optical coherence tomography (OCT) data using a longitudinal kidney donor and recipient cohort.

**Methods:**

We assessed 22 donors and 23 patients requiring renal transplantation over up to 1 year posttransplant. We measured choroidal thickness (CT) and area and compared our automated CT measurements to manual ones at the same locations. We estimated associations between choroidal measurements and markers of renal function (estimated glomerular filtration rate [eGFR], serum creatinine, and urea) using correlation and linear mixed-effects (LME) modeling.

**Results:**

There was good agreement between manual and automated CT. Automated measures were more precise because of smaller measurement error over time. External adjudication of major discrepancies was in favor of automated measures. Significant differences were observed in the choroid pre- and posttransplant in both cohorts, and LME modeling revealed significant linear associations observed between choroidal measures and renal function in recipients. Significant associations were mostly stronger with automated CT (eGFR, *P* < 0.001; creatinine, *P* = 0.004; urea, *P* = 0.04) compared to manual CT (eGFR, *P* = 0.002; creatinine, *P* = 0.01; urea, *P* = 0.03).

**Conclusions:**

Our automated approach has greater precision than human-performed manual measurements, which may explain stronger associations with renal function compared to manual measurements. To improve detection of meaningful associations with clinical endpoints in longitudinal studies of OCT, reducing measurement error should be a priority, and automated measurements help achieve this.

**Translational Relevance:**

We introduce a novel choroid segmentation algorithm that can replace manual grading for studying the choroid in renal disease and other clinical conditions.

## Introduction

Chronic kidney disease (CKD) is a major cause of morbidity and mortality, affecting over 800 million people worldwide.^1^ CKD is a gradual loss of kidney function, resulting in a failure to filter blood effectively and is associated with alterations in microvascular structure and function.^2^ Assessment of microvascular function is therefore helpful in the diagnosis, prognosis, and treatment of renal disease. Current clinical evaluation is based on blood and urine testing and invasive kidney biopsy.

The eye and the kidney show a striking resemblance in anatomy, physiology, and response to disease,^3^ and in particular, the choroidal microcirculation reflects the renal microcirculation.^4^ For example, they have an analogous vascular endothelium with similarly sized fenestrated vessel walls, allowing subretinal fluid exchange in the choroid and blood filtration in the kidneys. Moreover, the circulatory systems in the choroid and renal cortex have similar proportions of blood flow in contrast to their retinal and renal medullary counterparts.^5^

Choroidal thickness (CT) correlates strongly with renal dysfunction in patients with CKD compared with sex- and age-matched healthy and hypertensive individuals.^6^ This suggests that choroidal thinning may relate to systemic microvascular injury in general and renal injury in particular. An overactive sympathetic drive may contribute to disease progression in CKD^7^ and could explain choroidal thinning since choroidal perfusion is influenced by its autonomic supply.^6^ These observations suggest the potential clinical utility of choroidal biomarkers in renal disease. There is already a growing interest in choroidal biomarkers derived from optical coherence tomography (OCT) in nonocular pathology such as sepsis,^8^^,^^9^ diabetes and CKD,^6^^,^^10^^,^^11^ and neurodegenerative disease.^12^^–^^14^

Standard OCT imaging focuses on imaging the retina while enhanced depth imaging OCT (EDI-OCT) provides a deeper and stronger signal, visualizing the choroid–scleral (C-S) junction with micron resolution. Measuring the choroid manually from OCT images is a time-consuming and subjective process. Automated image analysis to extract choroidal measures as potential biomarkers could have substantial clinical utility if it is sufficiently reliable, robust, and reproducible. Consequently, there have been many attempts to validate automated algorithms for quantifying the choroid.^15^^–^^18^ However, only a handful of these proposed algorithms demonstrate their clinical utility in disease settings.^19^^,^^20^ To the best of our knowledge, such automated algorithms have rarely been applied to longitudinal image sets. This is important as accurate assessment of change over time is essential for tracking disease progression.

We have previously developed an automated approach to choroid region segmentation,^21^ and here we present our evaluation of its performance and clinical utility in a longitudinal cohort of individuals with end-stage CKD who underwent renal transplant and healthy donors who underwent unilateral nephrectomy. Our primary objective was to compare our automated CT measurements with the current gold standard of manual measurement of CT, as quantitative validation across longitudinal data is an ideal approach to evaluate novel image-processing methodologies in the medical domain.

As a secondary objective, we validate our segmentation algorithm clinically through estimating associations between the choroid and clinical biomarkers of renal function in both cohorts. In order to limit processing time, the choroid is typically measured manually using only thickness. Our approach permits automatic segmentation and subsequent calculation of choroidal thickness and area (CA), so we provide two metrics of the choroid to estimate associations with.

## Method

### Study Population

We prospectively analyzed a longitudinal cohort of healthy kidney donors and patients with end-stage kidney disease undergoing living donor kidney transplantation (NCT0213274).^22^ Data collection and subsequent analyses were conducted after ethical approval from the South East Scotland Research Ethics Committee, in accordance with the principles of the Declaration of Helsinki, and all participants gave informed consent to recruitment. Eligibility criteria for recruitment were (1) donors must be living and healthy throughout the period of analysis, (2) recipients with end-stage CKD have a functional kidney transplant, and (3) participants must be aged 18 years or over. To prevent ocular issues confounding our results, our exclusion criteria were (1) any ocular pathology pretransplant, (2) any previous eye surgery, (3) a refractive error exceeding ±6 diopters, or (4) a diagnosis of diabetes mellitus. We judged image quality by the OSCAR-IB criteria^23^ and excluded images with B-scan signal quality ≤15, indistinguishable C-S junction due to speckle noise, or partial image cropping of choroid.

Supplementary Figure S1 shows a flowchart on how the population was selected for this study. At the time of data extraction, 22 donors and 23 recipients were eligible for analysis, after excluding a total of 75 recipients and 5 donors. Most exclusions were from no eye scans being performed for these individuals. This was because, once recruited, they were scheduled for transplantation and then immediately returned to their referring institution after transplant away from Edinburgh, thus making it impractical to retain in the study and rescan. Each participant was assessed on the day of transplant, and then attempts were made at seven other time periods over the following year (eight time points in total).

Donors and recipients were organized into two data sets for *performance* evaluation and *clinical* evaluation. We used the entire analysis cohort for performance evaluation of the automated approach against manual grading, resulting in 483 CT measurements for comparison. After excluding two donors and seven recipients due to missing clinical measurements/eye scans posttransplant or poor-quality EDI-OCT images, we used 20 donors and 16 recipients for clinical evaluation (i.e., association estimation with clinical variables related to renal function). Clinical data at each time point included standard biomarkers of renal function, including high sensitivity C-reactive protein, serum urea and creatinine, estimated glomerular filtration rate (eGFR), and urine protein to creatinine ratio. Table 1 shows the population statistics for the entire analysis cohort.

**Table 1.** Population Statistics for All Donors and Recipients, Rounded to the Nearest Integer

	Baseline	1 Week	2 Weeks	4 Weeks	8 Weeks	18 Weeks	28 Weeks	52 Weeks
Demographic								
Donors								
Sample	22 (100)	11 (50)	2 (9)	8 (36)	10 (45)	8 (36)	10 (45)	11 (50)
Age, y	50 ± 11	—	—	—	—	—	—	—
Male sex	9 (41)	—	—	—	—	—	—	—
Approx. refractive error (D)	0.49 ± 1.17	—	—	—	—	—	—	—
Daytime (Hr:Min)	13:50 ± 1:41	13:48 ± 1:20	12:04 ± 1:34	12:48 ± 3:47	13:43 ± 2:12	13:54 ± 2:00	13:30 ± 2:03	13:48 ± 2:07
Time from baseline (wk)	—	1 ± 0	2 ± 0	4 ± 1	8 ± 2	16 ± 4	29 ± 3	53 ± 6
Recipients								
Sample	23 (100)	13 (61)	5 (48)	5 (48)	10 (43)	6 (26)	8 (35)	8 (35)
Age, y	47 ± 12	—	—	—	—	—	—	—
Male sex	15 (65)	—	—	—	—	—	—	—
Approx. refractive error (D)	−0.33 ± 1.73	—	—	—	—	—	—	—
Daytime (Hr:Min)	13:59 ± 2:19	12:44 ± 1:42	11:45 ± 1:50	12:23 ± 2:01	11:31 ± 1:13	12:29 ± 1:55	11:47 ± 1:39	12:25 ± 1:31
Time from baseline (wk)	—	1 ± 0	2 ± 0	4 ± 1	8 ± 2	17 ± 4	29 ± 4	52 ± 4
Clinical								
Donors								
BMI, kg/m^2^ (%)	27 ± 3 (95)	—	—	—	—	—	—	—
Systolic BP, mm Hg (%)	134 ± 14 (86)	—	—	—	—	—	—	—
Diastolic BP, mm Hg (%)	86 ± 18 (86)	—	—	—	—	—	—	—
MAP (%)	102 ± 15 (86)	—	—	—	—	—	—	—
hsCRP, mg/L (%)	1 ± 2 (59)	52 ± 30 (73)	2 ± 1 (100)	23 ± 34 (63)	1 ± 1 (70)	5 ± 9 (75)	1 ± 1 (80)	2 ± 4 (55)
Serum urea, mmol/L (%)	5 ± 1 (95)	6 ± 3 (91)	6 ± 1 (100)	6 ± 1 (88)	6 ± 1 (100)	7 ± 2 (75)	6 ± 1 (90)	6 ± 1 (64)
Creatinine, µmol/L (%)	69 ± 8 (95)	108 ± 25 (91)	102 ± 3 (100)	89 ± 7 (88)	102 ± 24 (100)	101 ± 25 (75)	100 ± 22 (90)	91 ± 9 (64)
eGFR, mL/min/1.73 m^2^ (%)	97 ± 11 (95)	61 ± 10 (91)	64 ± 9 (100)	71 ± 10 (88)	68 ± 18 (100)	67 ± 14 (75)	66 ± 12 (90)	67 ± 9 (64)
Urine P:Cr, mg/mmol (%)	3 ± 5 (41)	185 ± 303 (55)	1 ± 0 (50)	3 ± 3 (38)	1 ± 2 (60)	1 ± 2 (63)	1 ± 1 (60)	1 ± 1 (27)
Recipients								
BMI, kg/m^2^ (%)	27 ± 4 (87)	—	—	—	—	—	—	—
Systolic BP, mm Hg (%)	137 ± 14 (83)	—	—	—	—	—	—	—
Diastolic BP, mm Hg (%)	82 ± 8 (83)	—	—	—	—	—	—	—
MAP (%)	101 ± 9 (83)	—	—	—	—	—	—	—
hsCRP, mg/L (%)	3 ± 3 (48)	17 ± 20 (38)	3 ± 3 (40)	7 ± 10 (100)	3 ± 2 (30)	2 ± 2 (50)	1 ± 1 (38)	25 ± 52 (75)
Serum urea, mmol/L (%)	17 ± 8 (87)	7 ± 2 (77)	7 ± 2 (100)	6 ± 1 (100)	7 ± 2 (90)	7 ± 2 (83)	8 ± 2 (88)	6 ± 1 (88)
Creatinine, µmol/L (%)	581 ± 271 (87)	117 ± 35 (77)	92 ± 24 (100)	104 ± 25 (100)	116 ± 27 (90)	110 ± 32 (100)	115 ± 40 (88)	109 ± 27 (88)
eGFR, mL/min/1.73 m^2^ (%)	19 ± 28 (87)	61 ± 15 (77)	79 ± 16 (100)	71 ± 14 (100)	67 ± 20 (90)	70 ± 21 (100)	64 ± 23 (88)	69 ± 13 (88)
Urine P:Cr, mg/mmol (%)	295 ± 360 (48)	257 ± 28 (23)	84 ± 72 (40)	89 ± 57 (60)	93 ± 126 (50)	27 ± 14 (100)	34 ± 20 (63)	9 ± 13 (38)

Where appropriate, values are shown as mean ± SD, and values in parentheses represent proportion of data completeness relative to baseline populations. D, diopters; BMI, body mass index; BP, blood pressure; eGFR, estimated glomerular filtration rate; Hr:Min, hour:minutes; hsCRP, high-sensitivity C-reactive protein; MAP, mean arterial pressure; SD, standard deviation; Urine P:Cr, urine protein to creatinine ratio; wk, week; y, year.

### Image Acquisition

We imaged each participant's right eye using the Heidelberg spectral domain OCT Spectralis Standard Module (Heidelberg Engineering, Heidelberg, Germany). Patients were examined between 9 am and 5 pm and were endeavored to be followed up at around the same time of day at each time point. The average (and standard deviation) daytime in which scans were taken at each time point is listed in Table 1. During each patient's examination, a horizontal line, EDI-OCT B-scan centered at the foveal pit was taken. Each B-scan covered a 30° (8.7 mm) region and was extracted as a 768 × 768 (pixel height × width) high-resolution image for downstream image processing.

**Figure 1.**
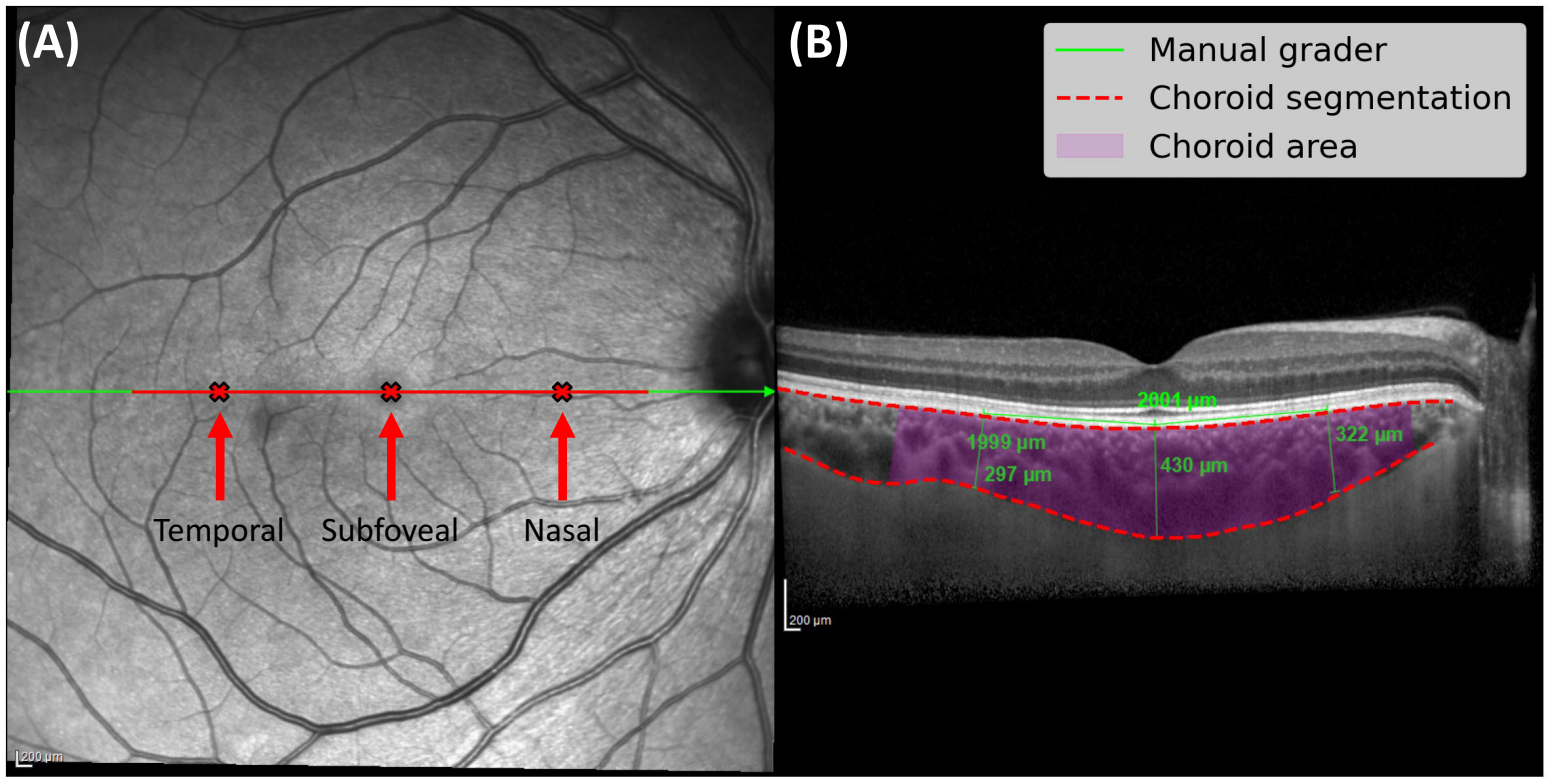
EDI-OCT scan of a donor's right eye at baseline. (**A**) En face view of the fundus with the location of the B-scan in *green* and markers for measuring CT and CA in *red*. (**B**) B-scan showing chorioretinal structures with manual and automated assessment shown.

Each scan was captured using active eye tracking with Automatic Real Time (ART) software built into the Spectralis device, averaging up to a maximum of 100 scans to generate a single high-resolution image. EDI-OCT images include the B-scan, alongside an en face view of the fundus with a horizontal line representing the B-scan's location on the macula. Although refractive error and axial length were not collected for this population, the built-in scan focus parameter (an approximate value for refractive error) was extracted from the acquisition metadata for each scan and was used for downstream clinical evaluation (listed in Table 1). Figure 1 shows an example EDI-OCT scan with results from manual and automated assessment. At each time point, only one EDI-OCT B-scan of each individual's right eye was selected for analysis, and no repeated scans were taken during the same examination.

### Manual Assessment of Choroidal Thickness

Manual grading for measuring CT was performed by a single, trained operator across all image sessions for each participant. Manual grading of the choroid is described in the original study carried out by Balmforth et al.^6^ Briefly, this was achieved using the caliper tool in HeyEx software (version 1.10.4.0; Heidelberg Engineering). Choroid thickness was measured at three locations across the macula—at the fovea and 2000 microns nasal and temporal to the fovea. The choroid region in each B-scan was defined as the area between the lower surface of the junction between the hyperreflective retinal pigment epithelium (RPE) layer and Bruch's membrane complex (RPE-C junction), and the upper surface of the junction between the choroid and sclera (C-S junction).

Manual grading used the caliper tools on the software to mark the center of the fovea at the level of the photoreceptor outer segment and mark 2000 microns temporal and nasal to this point, along the RPE-C junction. Choroid thickness was defined as the straight-line distance between the RPE-C and C-S junctions, measured locally *perpendicular* to the RPE-C junction. Figure 1 shows three CT measurements provided by the manual grader in green, including the reference lines measuring approximately 2000 microns temporal and nasal to the foveal pit along the RPE-C junction.

### Automated Assessment of Choroidal Thickness

**Figure 2.**
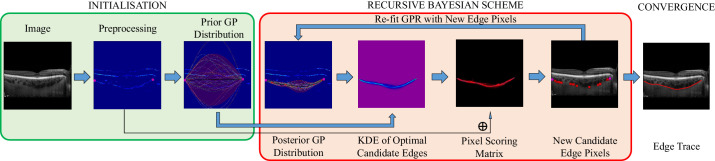
Schematic diagram of the automated approach segmenting the C-S junction of the choroid on an EDI-OCT B-scan.^21^ GP, Gaussian process; GPR, Gaussian process regressor; KDE, kernel density estimate.

Our automated choroid segmentation algorithm performed individual edge tracing to obtain the upper and lower choroid boundaries for each B-scan. The algorithm modeled each boundary using Gaussian process regression, building a posterior predictive function through a recursive Bayesian scheme and image gradient scoring mechanism to detect edge pixels.^21^ Figure 2 presents a schematic diagram of the image analysis pipeline of the automated approach. Preprocessing was done using median filter denoising and contrast enhancement using contrast-limited adaptive histogram equalization. Approximate edge maps were then computed using discrete derivative and morphologic erosion and dilation filters.

Automated CT was measured in two different ways, yielding *perpendicular* CT measurements and *parallel* CT measurements. *Perpendicular* automated CT measured locally *perpendicular* to the RPE-C junction, while *parallel* automated CT measured parallel to the manual measurements to mimic any potential angular error made by the manual grader. See, for example, the nasal measurement in Figure 5C, where perpendicular automated CT is shown in red, parallel automated CT in blue, and manual CT in green. We measured CT in the same subfoveal, temporal, and nasal locations as manual grading. We measured CA by computing the area of pixels within a 3000-micron radius centered at the foveal pit, in accordance with the Early Treatment Diabetic Retinopathy Study area of 6000 × 6000 microns.^24^

### Statistical Analysis

#### Performance Evaluation

We measured agreement between approaches by comparing (parallel) automated CT to manual CT and performed Passing–Bablok analysis^25^ to determine any systematic or proportional differences between the two approaches. We also investigated residuals, calculated by subtracting manual CT from automated CT (automated – manual), using a Bland–Altman plot.^26^ Mean absolute error (MAE), Pearson correlation and intra-class correlation were also computed between both approaches, stratified by cohort and macular location. To compare the consistency of measurements across longitudinal data, we investigated the angular deviation made by the manual grader. This was done by computing angles between the caliper measurements made from manual grading against the perpendicular automated CT measurements. Automated assessment of the choroid and quantitative analyses comparing manual and automated measurements were done in Python (version 3.11.3).

Rahman et al.^27^ found an unsigned residual of 32 µm as a threshold suggested to exceed interobserver variability between graders of EDI-OCT images. Therefore, we defined major discrepancies between automated and manual CT to be any residual greater than 32 µm in absolute value. These were selected for external adjudication by a clinical ophthalmologist, I.M. For each discrepancy, I.M. was shown two CT measurements displayed on the corresponding choroid and was blinded to which approach was used in each case. He was asked to rate each measurement and the overall visibility of the C-S junction using an ordinal scale: “bad,” “okay,” and “good.” I.M. was also asked to select the approach that was preferred—options for both or neither approaches were also available.

#### Clinical Evaluation

We investigated how CT and CA measurements changed over time in donors and recipients, and estimated linear associations with clinical variables related to renal function using Pearson correlations—specifically eGFR, serum creatinine, and serum urea. Other clinical measures were too incomplete and inconsistently collected to be analyzed with the current sample size. CT was averaged across macular locations, resulting in a single value of thickness for each scan. We examined the difference in CT and CA between measurements at baseline and 1 year posttransplant, testing for difference in their means longitudinally via the Student's *t*-test for dependent, paired samples. We specified a *P* value of 0.05 as the threshold for statistical significance.

We also looked for significant linear associations between markers of renal function and the choroid. We assumed a nested structure to the data and used linear mixed-effects (LME) models, setting an individual random effect to model the variation among individuals, accounting for age, sex, approximate refractive error (measured in diopters), and the time of day the scan was taken, relative to the time of day their baseline scan was taken (measured in decimal hours). For each clinical measure of renal function, we fitted an LME model for each choroidal image measure in turn as one of the associated independent variables. LME modeling was carried out using R (version 4.2.2).^28^ We identify independent variables as statistically significant predictors if their associated 95% confidence interval excludes 0.

## Results

### Performance Evaluation With Choroidal Thickness

Performance results between automated and manual CT measurements are shown in Table 2. There was good agreement between manual and automated measures of CT, with an average residual of 1.8 ± 22.0 µm and MAE of 14.1 ± 16.9 µm across all 483 CT measurements. Moreover, Pearson and intraclass correlation show excellent correlations between both approaches. The largest residuals were at the subfoveal macular location.

**Table 2.** Performance Evaluation Between Automated and Manual CT, Stratified by Cohort and Macular Location

		Donors			Recipients		
Metric	All	Temporal	Subfoveal	Nasal	Temporal	Subfoveal	Nasal
Residual CT (µm)	1.8 ± 22.0	5.1 ± 18.4	−5.9 ± 17.4	2.1 ± 18.8	2.9 ± 24.3	3.0 ± 28.1	3.8 ± 21.0
MAE CT (µm)	14.1 ± 16.9	13.8 ± 13.2	13.4 ± 12.5	12.9 ± 13.8	13.5 ± 20.4	17.3 ± 22.3	13.8 ± 16.2
Pearson, rp	0.97	0.96	0.98	0.97	0.92	0.94	0.96
ICC (3,1)	0.96	0.95	0.97	0.96	0.92	0.94	0.96

Where appropriate, values are shown as mean ± SD. All Pearson and intraclass correlations were statistically significant. ICC, intraclass correlation.

**Figure 3.**
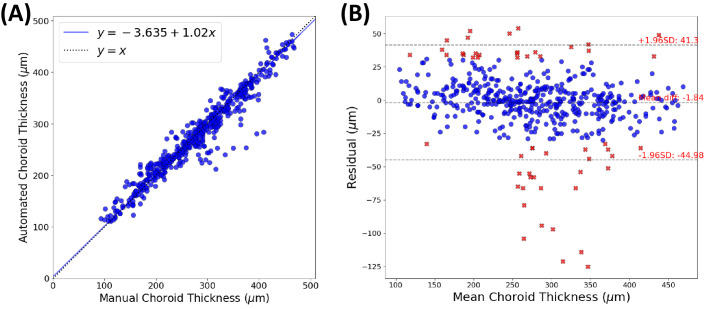
(**A**) Correlation plot of manual and (parallel) automated CT measurements. (**B**) Bland–Altman plot showing residual values, with 52 major discrepancies highlighted as *red crosses*.

Passing–Bablok analysis computed a slope value of 1.02 (95% confidence interval [CI], 0.99–1.04) and an intercept of −3.64 µm (95% CI, −10.78 to 3.50 µm) with an *R*^2^ value of 0.93 (Fig. 3A). These 95% confidence intervals include 1 and 0, respectively, suggesting that the automated and manual approaches are equivalent.^25^^,^^29^ See Supplementary Figures S2 and S3 for correlation plots stratified by macular location and cohort. Bland–Altman analysis in Figure 3B computed an average residual of 1.84 µm with limits of agreement of −44.98 to 41.30 µm. Only 10.78% of all 483 CT measurements (52/483) exceeded an unsigned residual of 32 µm.^27^

Table 3 presents the results from the external adjudication of all 52 major discrepancies. Overall, the automated CT measurements scored higher in terms of preference, where 79% (41/52) of the automated thicknesses were preferred compared to 58% (30/52) for manual thicknesses. Moreover, the automated approach scored higher in terms of quality, with only 1 measurement judged as “bad” versus 10 measurements judged “bad” for the manual approach. Figure 5 shows four major discrepancies from four different choroids. Figure 5A shows the largest observed discrepancies—temporal (97 µm) and subfoveal (114 µm)—with adjudicator I.M. preferring the automated measurements.

**Table 3.** Numerical Score of Both Approaches from Masked Adjudication of 52 Major Discrepancies, Stratified by the Visibility of the C-S Junction, and Qualitative Preference Scores for Both Approaches

	Score	
C-S Junction Visibility	Automated	Manual
Good (*n* = 9)	8	2
Okay (*n* = 15)	9	13
Bad (*n* = 28)	24	15
Total (*N* = 52)	41	30
Method	Discrepancy Measurement	
Automated	Good: 32, Okay: 19, Bad: 1	
Manual	Good: 24, Okay: 18, Bad: 10	

**Figure 4.**
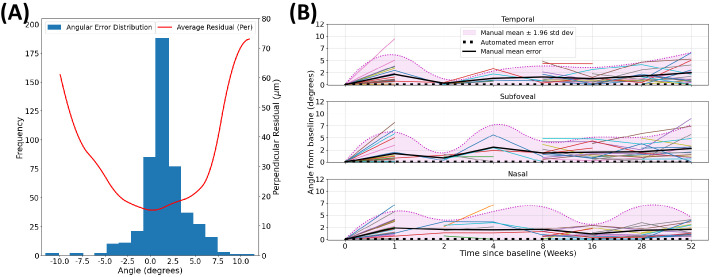
(**A**) Distribution of angular errors from manual CT measurements (*blue*). Average unsigned difference between manual and perpendicular automated CT (*red*). (**B**) Unsigned, longitudinal within-patient angular deviations, relative to baseline measurements. *Colored lines* represent distinct individuals using manual measurements.

We found that deviation in the angle of manual measurement from perpendicular led to a disproportionate error between perpendicular automated CT and manual CT. This is illustrated in Figure 4A, which shows the distribution (mean 0.5 and standard deviation 2.5) of angular errors made from manual grading. Absolute differences between manual CT and perpendicular automated CT grew quadratically as the angular error deviated from 0. We also found large deviation in manual grading when tracking the longitudinal, within-patient angular difference from baseline (Fig. 4B). Distributions at each time point were estimated through empirical mean and standard deviation values. Note that the mean fluctuation in manual grading was always above 0 in comparison to the mean fluctuation from the automated approach, which was constant at 0.

**Figure 5.**
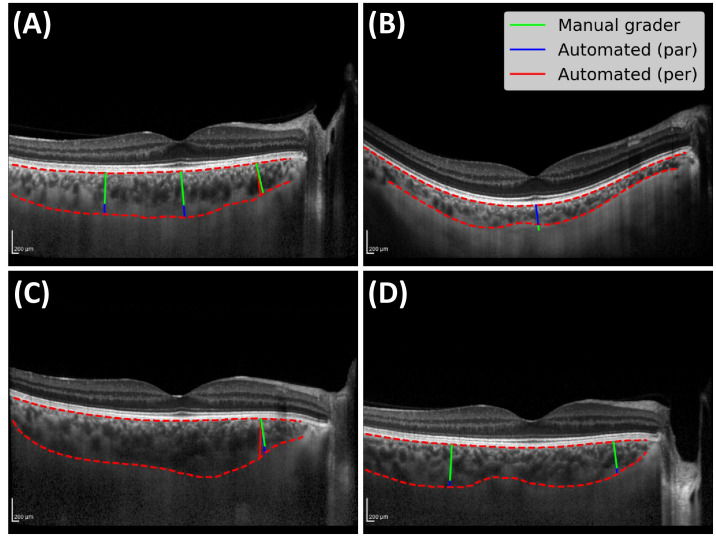
(**A**) A selection of major discrepancies between automated and manual CT measurements. *Red lines* represent perpendicular automated CT, *blue* represent parallel automated CT, and *green* represent manual CT. The lines in *red* are shown in (**A**, **C**) to demonstrate the observed angular deviation between perpendicular automated CT and manual CT. (**A**) Temporal and subfoveal major discrepancies from a donor, residuals 97 µm and 114 µm, respectively. Nasal angular deviation (5.4°) resulted in perpendicular and parallel residuals as 22 µm and 2 µm, respectively. (**B**) Subfoveal major discrepancy from a recipient, residual –47 µm. (**C**) Nasal angular deviation (8.1°) shows perpendicular and parallel residuals as 106 µm and 79 µm, respectively. (**D**) Temporal and nasal major discrepancy from a donor, residuals 66 µm and 65 µm, respectively.

### Choroidal Association With Renal Function

**Figure 6.**
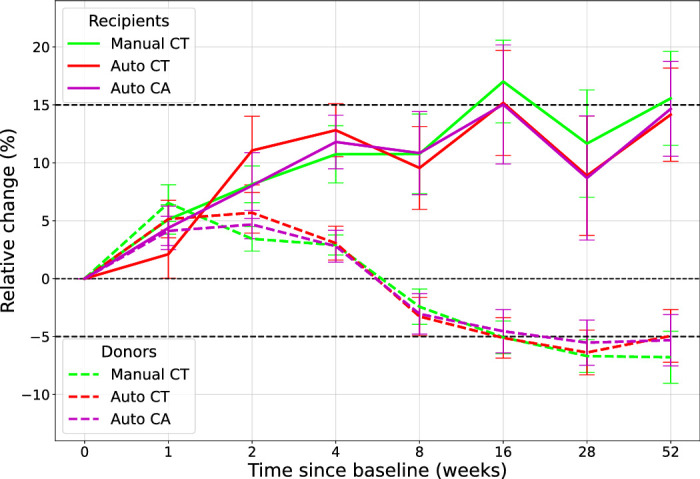
Longitudinal change in the choroid, measured relative to baseline measurements. Donors are shown as *dashed lines* and recipients as *solid lines*, with standard errors plotted at each time point.

All measures of renal function and choroidal measurements were significantly different 1 year posttransplant compared to baseline measurements in both cohorts (Table 4). Figure 6 shows the average percentage change in choroidal measurements from baseline measurements in both cohorts. In transplant recipients, there was a significant increase in choroidal thickness of 12.8% ± 4.8% (median, 11.4%) from baseline after 4 weeks posttransplant using automated CT (*P* = 0.02). After 1 year posttransplant, we observed a significant increase of 14.1% ± 11.4% (median, 10.7%) from baseline (*P* = 0.01). Interestingly, we observed a significant increase of 5.1% ± 5.6% (median, 5.4%) 1 week posttransplant in the choroid of donors (*P* = 0.01). The average choroid remained inflated relative to baseline measurements, and at some point between 4 and 8 weeks posttransplant, the choroid deflated. After 1 year posttransplant, we observed a significant decrease of −4.9% ± 7.9% (median, –3.5%) in automated CT (*P* = 0.01).

**Table 4.** Choroidal Measurements at Baseline and 1 Year Posttransplant (PT)

	Donors			Recipients		
Measurement	Baseline	1 Year PT	*P* Value	Baseline	1 Year PT	*P* Value
Automated CA (mm^2^)	1.59 ± 0.41	1.49 ± 0.41	0.01	1.58 ± 0.41	1.74 ± 0.46	0.01
Automated CT (µm)	278 ± 69	259 ± 68	0.01	271 ± 70	301 ± 75	0.01
Manual CT (µm)	278 ± 61	258 ± 62	0.006	262 ± 64	292 ± 66	0.008
eGFR (mL/min/1.73 m^2^)	91 ± 5	64 ± 7	<0.001	8 ± 3	69 ± 14	<0.001
Creatinine (mol/L)	69 ± 9	92 ± 10	<0.001	659 ± 196	109 ± 29	<0.001
Urea (mmol/L)	5.0 ± 0.8	6.8 ± 1.3	0.002	18.5 ± 9.7	6.7 ± 1.7	0.005

*P* values calculated using Student's dependent *t*-test for related samples.

In the recipient cohort, all choroidal measurements linearly correlated with eGFR, serum creatinine, and serum urea with statistical significance. All Pearson correlation coefficients with renal function were stronger in automated choroid measurements than in manual ones (Table 5). There were poor and insignificant linear correlations found between the choroid and renal function in the donor cohort, as seen in Supplementary Table S1, which also shows the corresponding *P* values for all correlation analyses performed.

**Table 5.** Pearson Correlation Coefficients Between Choroidal Measurements and eGFR, Serum Creatinine, and Serum Urea in the Recipient Cohort

	Pearson, rp		
Image Measure	eGFR	Creatinine	Urea
Automated CA	0.72	−0.77	−0.73
Automated CT	0.81	−0.76	−0.74
Manual CT	0.69	−0.76	−0.72

All correlations presented here were significant such that *P* < 0.05 apart from eGFR using manual CT.

Using all choroidal measurements, in transplant recipients, LME modeling showed evidence of a statistically significant, positive linear association with eGFR and negative associations with serum creatinine and urea levels. Table 6 shows the model summary's for predicting eGFR, serum creatinine, and serum urea using each choroidal measurement in turn. Models using automated measures had greater conditional *R*^2^ values—a measure analogous to the *R*^2^ value for linear models but taking into account the random- and fixed-effects variables—and their respective independent variables showed stronger association to predicting renal function. Interestingly, the time difference between follow-up and baseline image acquisition was a statistically significant predictor of all markers of renal functions for models using all choroidal metrics.

**Table 6.** LME Summary Tables for Predicting eGFR, Serum Creatinine, and Serum Urea in Transplant Recipients Using Each Choroidal Measurement

	eGFR			Creatinine			Urea		
	β	95% CI	*P* Value	β	95% CI	*P* Value	β	95% CI	*P* Value
Automated choroid area									
Intercept	−0.1	(−1.38, 1.17)	0.84	0.09	(−1.18, 1.36)	0.86	0.03	(−0.49, 0.55)	0.9
Age	0.98	(−0.47, 2.43)	0.15	−1.11	(−2.54, 0.31)	0.1	−0.3	(−0.95, 0.34)	0.29
Sex (male)	−0.24	(−1.55, 1.07)	0.66	0.27	(−1.03, 1.57)	0.6	0.22	(−0.31, 0.76)	0.34
**Daytime from** **baseline**	−0.4	(−0.59, −0.21)	**≤0.001**	0.59	(0.39, 0.80)	**≤0.001**	0.57	(0.32, 0.81)	**≤0.001**
Approx. refraction	−0.86	(−2.17, 0.46)	0.114	0.92	(−0.37, 2.21)	0.12	0.31	(−0.28, 0.89)	0.24
**Automated CA**	1.54	(0.97, 2.10)	**≤0.001**	−1.52	(−2.12, −0.92)	**≤0.001**	−0.6	(−1.09, −0.11)	**0.02**
Patient SD	1.63	Conditional *R*^2^: 0.93	0.6	Conditional *R*^2^: 0.91	0.55	Conditional *R*^2^: 0.57			
Automated choroid thickness									
Intercept	−0.1	(−1.19, 0.99)	0.83	0.07	(−0.91, 1.05)	0.84	0.03	(−0.48, 0.53)	0.9
Age	0.74	(−0.51, 1.99)	0.2	−0.8	(−1.92, 0.31)	0.12	−0.27	(−0.90, 0.36)	0.33
Sex (male)	−0.31	(−1.43, 0.81)	0.49	0.31	(−0.69, 1.30)	0.44	0.28	(−0.25, 0.81)	0.24
**Daytime from** **baseline**	−0.46	(−0.67, −0.24)	**≤0.001**	0.65	(0.42, 0.88)	**≤0.001**	0.55	(0.30, 0.80)	**≤0.001**
Approx. refraction	−0.43	(−1.55, 0.68)	0.37	0.46	(−0.54, 1.46)	0.27	0.18	(−0.35, 0.71)	0.42
**Automated CT**	1.15	(0.51, 1.78)	**≤0.001**	−1.01	(−1.62, −0.40)	**0.004**	−0.54	(−1.06, −0.02)	**0.04**
Patient SD	1.37	Conditional *R*^2^: 0.88	1.05	Conditional *R*^2^: 0.81	0.5	Conditional *R*^2^: 0.52			
Manual choroid thickness									
Intercept	−0.1	(−1.22, 1.02)	0.83	0.07	(−0.89, 1.03)	0.84	0.03	(−0.47, 0.52)	0.9
Age	0.7	(−0.59, 1.98)	0.23	−0.72	(−1.82, 0.38)	0.14	−0.27	(−0.89, 0.35)	0.33
Sex (male)	−0.29	(−1.44, 0.86)	0.55	0.27	(−0.71, 1.24)	0.48	0.28	(−0.24, 0.80)	0.23
**Daytime from** **baseline**	−0.47	(−0.68, −0.25)	**≤0.001**	0.66	(0.43, 0.90)	**≤0.001**	0.56	(0.31, 0.80)	**≤0.001**
Approx. refraction	−0.41	(−1.56, 0.74)	0.41	0.41	(−0.57, 1.39)	0.29	0.18	(−0.35, 0.70)	0.42
**Manual CT**	1.11	(0.45, 1.76)	**0.002**	−0.89	(−1.54, −0.24)	**0.01**	−0.56	(−1.06, −0.05)	**0.03**
Patient SD	1.39	Conditional *R*^2^: 0.87	0.99	Conditional *R*^2^: 0.79	0.49	Conditional *R*^2^: 0.52			

Results for the donor cohort are shown in Supplementary Table S2 but were omitted due to a lack of significant results with the choroid. β represent standardized model coefficients, with their corresponding 95% confidence intervals and associated *P* values. Statistically significant variables are shown in bold, as well as their corresponding *P* values.

There were no statistically significant associations found between any choroidal measurement and markers of renal function in the donor cohort. However, sex was associated with predicting serum creatinine using automated choroidal measurements, with weak statistical significance. These LME results can be found in Supplementary Table S2.

## Discussion

We found that automated CT measurements agreed well with manual ones in general but have greater precision, especially when applied to a longitudinal series of images. Much of the superior precision from automated CT results from eliminating inconsistent measurement angle, since the automated method is always able to measure CT locally perpendicular at the exact same location across all time points for each patient—this is an important attribute for measurement protocol so that we can account for choroidal curvature, or if the choroid does not appear predominantly horizontal in the image. In contrast, error in the angle of manual measurement had a disproportionate impact.

The RPE-C junction is almost never parallel to the C-S junction, making it a challenge for humans to measure perpendicular angles by eye across repeated measures, which is a major source of error. Furthermore, the different axial and lateral resolutions in each B-scan can result in the slightest change in marking a lateral position during manual grading to misrepresent the true CT. Figures 5A and 5C represent two exemplar cases, where the residuals between manual CT and perpendicular/parallel automated CT measurements are very different.

Importantly, the superior precision of the automated method translated into stronger associations with renal function, at least in the recipient cohort, where statistical significance was observed. This illustrates the necessity for reliable and reproducible measurements in longitudinal studies and their subsequent impact on predicting clinical outcomes.

Much of the discrepancy between manual and automated CT arose from choroidal features that are inherently difficult to measure. For example, the C-S junctions of larger choroids (Figs. 5A, 5C, 5D) are prone to poor-quality image acquisition due to a higher incidence of poor signal penetration. However, the external adjudicator preferred the automated measurements over the manual grader for these major discrepancies. Furthermore, the C-S junction may be obscured in choroids whose posterior of Haller's layer is low contrast (Fig. 5B)—here, the external adjudicator preferred the manual grader's measurement. The choroid in Figure 5A represents a source of major disagreement between the manual grader and the automated approach. The manual grader has defined the C-S junction as the boundary below the most visible posterior vessels (green), while the automated approach has successfully identified vessels with much lower visibility further below the clearer vessels (red), of which the external adjudicator agreed with.

The choroids in Figure 5 represent just over half (51.9%) of all major discrepancies lying outside the threshold suggested to exceed interobserver variability between graders of EDI-OCT images.^27^ That is, most outliers came from the same four choroids at different time points. Moreover, of the 28 thickness measurements from choroids described as having poor visibility, 20 of them came from the larger choroids (Figs. 5A, 5C, 5D).

From both correlation analysis and LME modeling, automated choroidal measurements were found to correspond with markers of renal function over time better than manual CT, probably because of reduced measurement error. All choroidal measurements changed substantially over time for all study participants, as did eGFR, serum creatinine, and serum urea. This is consistent with the choroid reflecting renal function during treatment of CKD. The choroid, and indeed CT/CA, does vary naturally during the day due to diurnal variation. Tan et al.^30^ describe the average change in CT across daytime hours to be approximately 8.5% ± 5.2%. The change in automated CT in transplant recipients 1 year posttransplant was 14.1% ± 11.4% (14.7% ± 11.2% for CA). This suggests that choroidal inflation in transplant recipients cannot be explained fully through diurnal variation but potentially through improved renal function.^31^ However, the same statement for healthy kidney donors cannot be made as confidently, where we only observed an average decrease in CT of −4.9% ± 7.9% (−5.3% ± 7.7% for CA).

We must note that big fluid shifts occur at the time of transplantation, although we have not been able to account for any change in body fluid volume posttransplant and what impact that may have had on the choroid. However, these fluid shifts would only impact the choroid in the short term, and our follow-up data are of sufficient time course to exclude any potential long-term effect from body fluid volume on the significant changes we see in the choroid.

In each LME model, we accounted for age, sex, approximate refractive error, and the intraparticipant relative-to-baseline daytime each scan was taken (relative daytime). The latter two measurements were used to assess the impact of myopia and diurnal variation in both cohorts for predicting renal function using the choroid. Relative daytime proved to be a significant predictor variable for markers of renal function alongside all choroidal measurements, while approximate refractive error did not. Note, however, that the standardized model coefficients are almost always larger in absolute value for the choroidal measurements versus relative daytime.

In every model, there was an opposite effect of relative daytime compared with the choroid, suggesting that the size of the choroid is negatively offset such that the CT and CA in a follow-up scan taken later in the day (relative to the baseline acquisition time) are decreased in absolute value—and vice versa for follow-up scans taken earlier than the original baseline scan. This makes sense given we know that the choroid decreases marginally through the course of the day.^30^

This suggests that while choroidal measurements still play a significant role in predicting markers of renal function for transplant recipients, it is important that daytime be recorded for downstream analysis, and image acquisition try be performed around the same time of day so as not to diminish any longitudinal signal present in the data. Note, however, that removing relative daytime still resulted in the choroid as a statistically significant predictor variable, but the overall model fit was marginally worse (data not shown).

It may be that the immediate inflation of the choroid in the first 4 weeks after kidney donation in donors could be a result of a prompt systemic, cardiovascular response to the unilateral nephrectomy. While from 8 weeks posttransplant, we observed choroidal thinning from all measurements, consistent with previous literature,^32^ it is within the first month in a donor's recovery period that compensatory renal hypertrophy takes place.^33^^,^^34^ These observations align with Choi and Kim,^31^ who cautiously suggest that a decrease in subfoveal CT is associated with a decline in renal function, but its increase is associated with renal hypertrophy, albeit in patients with diabetic retinopathy.

Low signal quality, vessel shadowing, lack of signal penetration, and the potential for extravascular fluid to obscure and occlude the boundary all contribute to the challenging task of tracing the C-S junction in OCT images. However, the automated segmentation methodology has the ability to overcome many of these problems due to its tunable hyperparameters and model uncertainty quantification.^21^ For example, the Bayesian recursive scheme allows uncertainty in obscured or occluded regions to be quantified and correctly interpolated. This feature is advantageous for choroid region segmentation. Moreover, the methodology provides an analytical, functional form for the edge that is explainable, interpretable, and reproducible—features that tend to be absent from many deep learning algorithms in medical image segmentation.^35^

There were a number of limitations in this study. First, our two cohorts were limited in sample size, and not all participants or clinical variables were collected at every time point. Moreover, of the 20 donors and 16 recipients included for clinical evaluation, only 11 donors and 8 recipients were followed up 1 year posttransplant, which potentially introduced bias into our clinical evaluation and contributed to a reduction of statistical power to which we could draw our conclusions with. Furthermore, using a subcohort for clinical evaluation could potentially introduce selection bias when estimating associations between the choroid and renal function. Therefore, further studies with a larger and more complete data set would permit a higher confidence regarding the significance of our analyses. However, while the results of the clinical evaluation may suffer from a lack of complete image or clinical data, our primary objective of comparing manual CT with automated CT remains undiluted by these limitations.

Another limitation is that the automated approach does require some, albeit minimal, form of manual intervention to select the edge endpoint pixels before tracing each boundary of the choroid in a single B-scan. Another limitation is the possibility of underapproximating the C-S junction for choroids such as the one in Figure 5B. Finally, while we have provided an analysis on regional quantities of the choroid and their potential links to microvascular injury and renal function, further work would aim to quantify any relationships with the vasculature within the choroid. This could be done, for example, by computing the choroid vasculature index, which has recently become a popular and more robust way to characterize the choroid for ocular and nonocular pathology.^11^^,^^36^^–^^38^

## Conclusions

Our automated segmentation methodology can replace human measures for studying the choroid in renal disease and potentially other clinical conditions. We observed no systematic or proportional differences when comparing manual CT to automated CT measurements. While the automated approach agreed strongly with manual ones, automated CT eliminated angular error and therefore had greater precision when assessing within-patient longitudinal data. The error in manual measurement ultimately led to a lack of strength and significance in their clinical associations. High precision and reproducibility of image measures are critical for longitudinal studies of the choroid where repeated measurements are made using highly sensitive retinal imaging modalities for predicting clinical outcomes.

We observed significant choroidal thickening in recipients 1 year posttransplant, and the choroid corresponded to improved eGFR, serum creatinine, and urea levels. In donors, we observed choroidal inflation in the first 4 weeks after the unilateral nephrectomy, followed by significant thinning 1 year posttransplant. Initial inflation of the choroid could possibly be related to compensatory renal hypertrophy, while long-term thinning may be linked to long-term impairment of renal function. Future work should aim at a fuller analysis with a larger and more complete data set in order to increase statistical power.

Our general-purpose segmentation methodology is freely available on GitHub,^39^ and we are working on producing an automated, end-to-end, and open source framework for choroidal image analysis in OCT data. Automated tools to assist clinical prognosis and treatment will strengthen future clinical studies, enabling the delivery of robust, reproducible, and responsible research in areas that have previously been at risk of human error.

## Supplementary Material

Supplement 1

Supplement 2

Supplement 3

Supplement 4

Supplement 5
