# Supplementary material for: Evaluation of an Automated Choroid Segmentation Algorithm in a Longitudinal Kidney Donor and Recipient Cohort
Source: Transl Vis Sci Technol. 2023 Nov 17;12(11):19. doi: 10.1167/tvst.12.11.19 (PMC10668611; doi:10.1167/tvst.12.11.19)
Supplement: Supplement 1 [file tvst-12-11-19_s001.pdf]

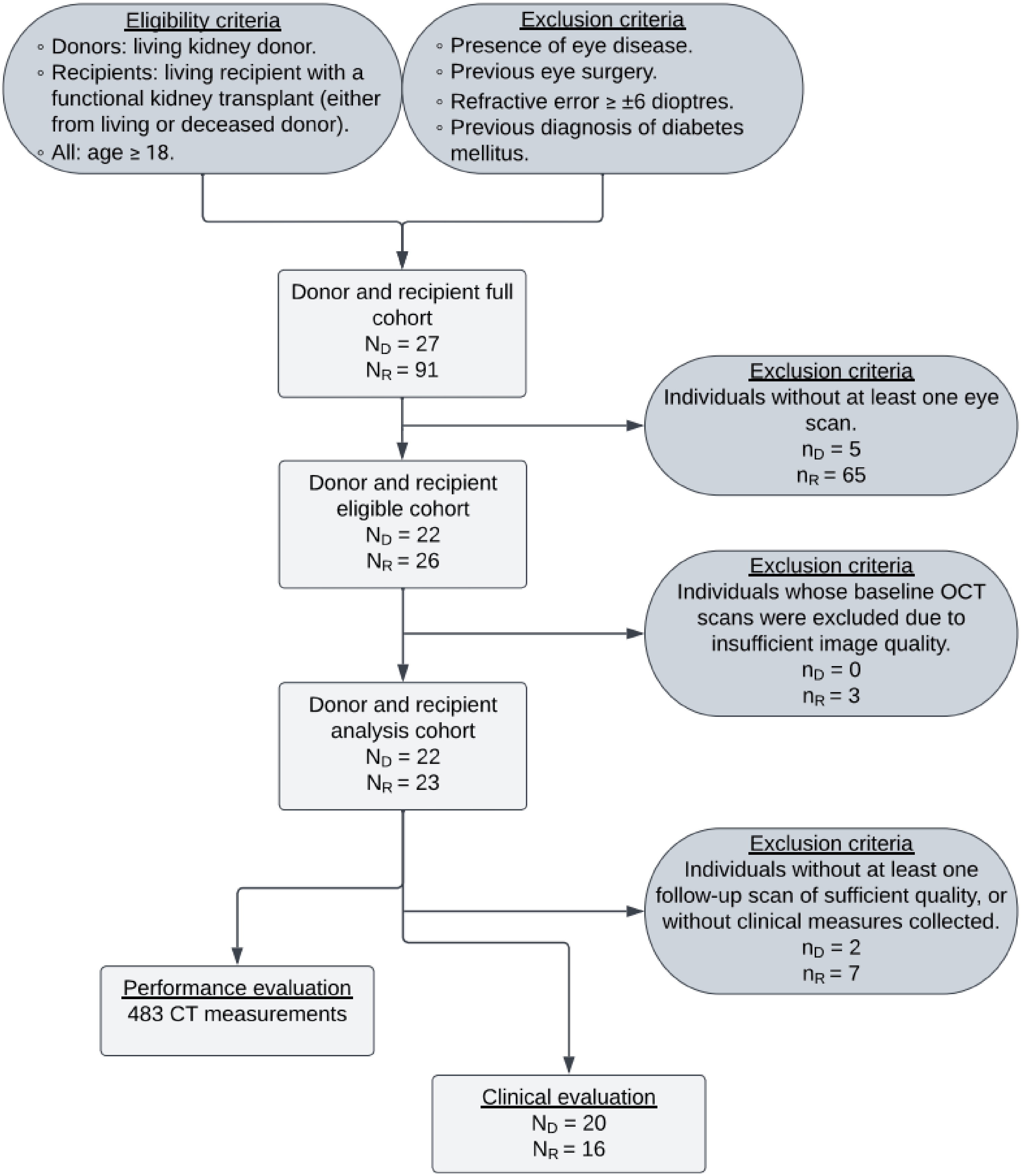

Figure S1: Flow chart visualising donor and recipient cohort specification. N_D and N_R represent number of donors and recipients included at each level. n_D and n_R represent number of donors and recipients excluded at each level, respectively.
