# Supplementary material for: Evaluation of an Automated Choroid Segmentation Algorithm in a Longitudinal Kidney Donor and Recipient Cohort
Source: Transl Vis Sci Technol. 2023 Nov 17;12(11):19. doi: 10.1167/tvst.12.11.19 (PMC10668611; doi:10.1167/tvst.12.11.19)
Supplement: Supplement 2 [file tvst-12-11-19_s002.pdf]

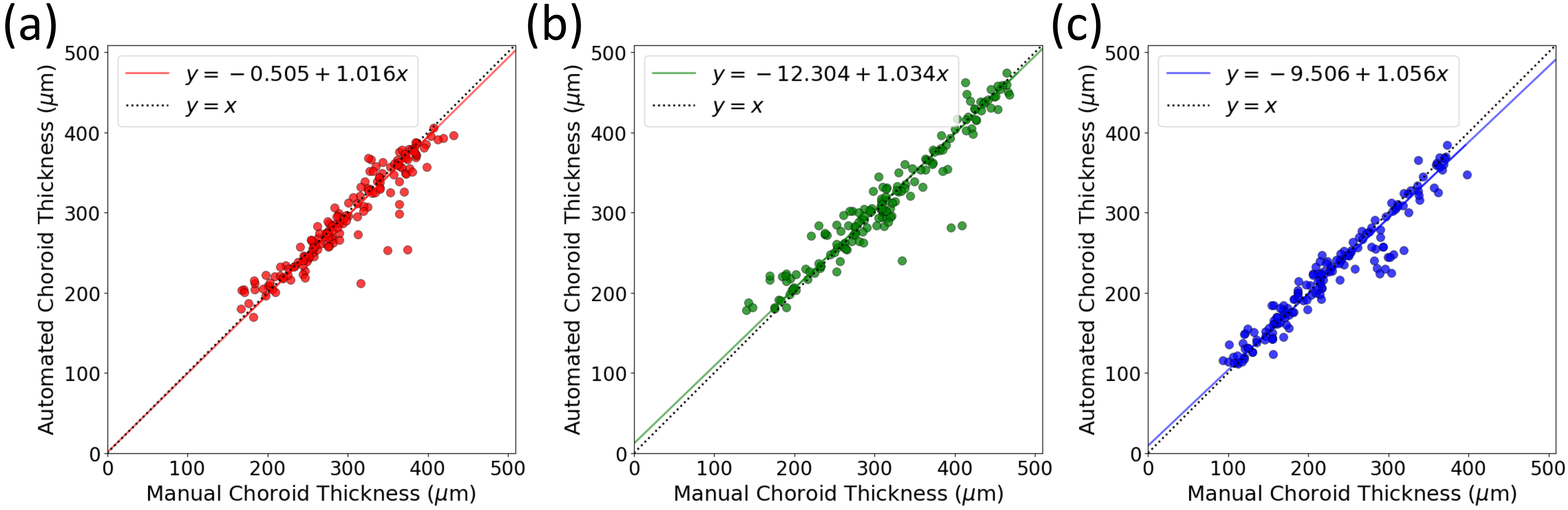

Figure S2: Correlation plot comparing manual and automated choroid thickness measurements, stratified by macular location; temporal (a), subfoveal (b), nasal (c).
