# Supplementary material for: Evaluation of an Automated Choroid Segmentation Algorithm in a Longitudinal Kidney Donor and Recipient Cohort
Source: Transl Vis Sci Technol. 2023 Nov 17;12(11):19. doi: 10.1167/tvst.12.11.19 (PMC10668611; doi:10.1167/tvst.12.11.19)
Supplement: Supplement 3 [file tvst-12-11-19_s003.pdf]

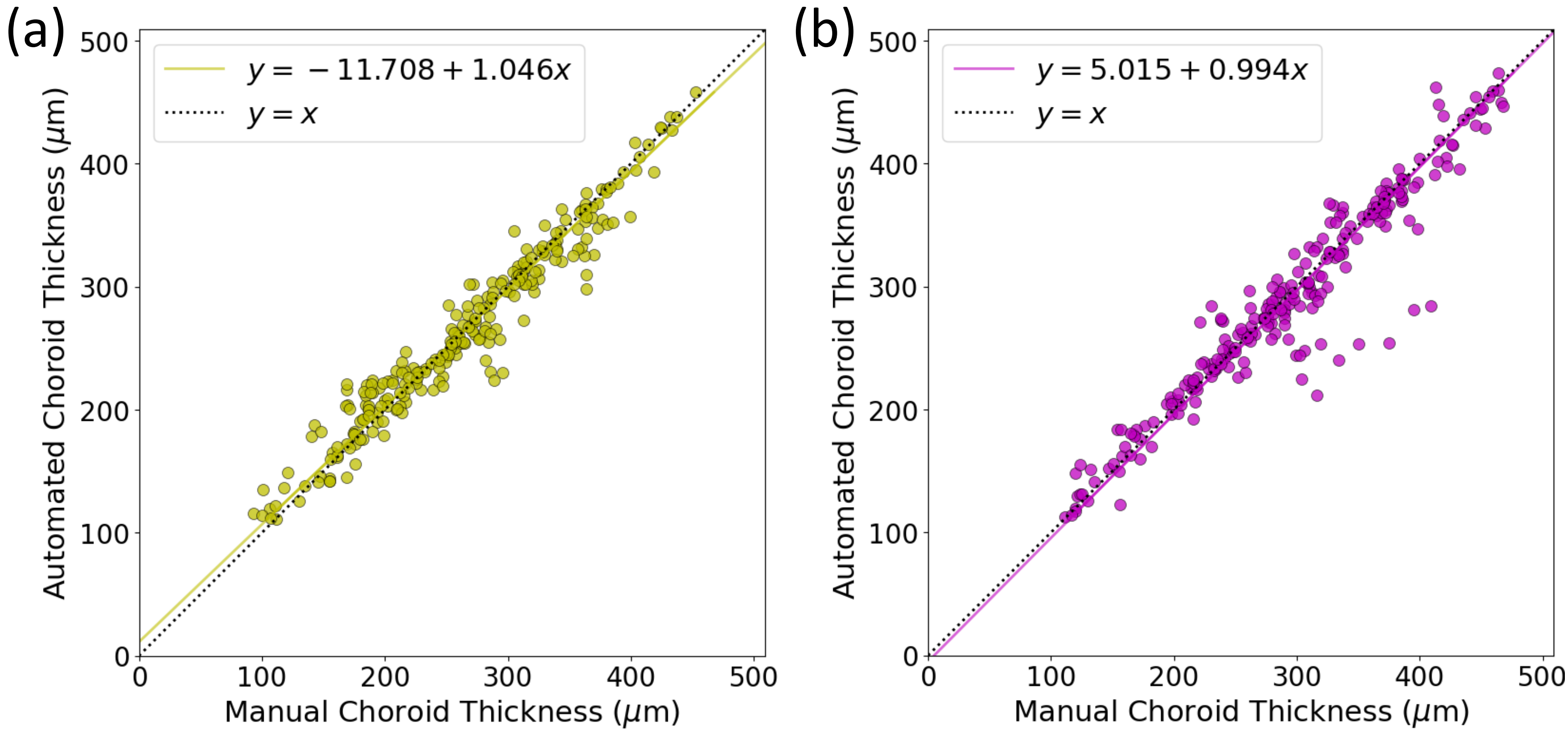

Figure S3: Correlation plot comparing manual and automated choroid thickness measurements, stratified by cohort; donors (a), recipients (b).
